# Investigation of Tumor Cell Behaviors on a Vascular Microenvironment-Mimicking Microfluidic Chip

**DOI:** 10.1038/srep17768

**Published:** 2015-12-03

**Authors:** Rong Huang, Wenfu Zheng, Wenwen Liu, Wei Zhang, Yunze Long, Xingyu Jiang

**Affiliations:** 1 College of Physics & Collaborative Innovation Center for Marine Biomass Fibers, Materials and Textiles of Shandong Province, Qingdao University, No. 308 Ningxia Road, Qingdao 266071, China; 2 Beijing Engineering Research Center for BioNanotechnology & CAS Key Laboratory for Biological Effects of Nanomaterials and Nanosafety, National Center for NanoScience and Technology, 11 BeiYiTiao, ZhongGuanCun, Beijing 100190, China

## Abstract

The extravasation of tumor cells is a key event in tumor metastasis. However, the mechanism underlying tumor cell extravasation remains unknown, mainly hindered by obstacles from the lack of complexity of biological tissues in conventional cell culture, and the costliness and ethical issues of *in vivo* experiments. Thus, a cheap, time and labor saving, and most of all, vascular microenvironment-mimicking research model is desirable. Herein, we report a microfluidic chip-based tumor extravasation research model which is capable of simultaneously simulating both mechanical and biochemical microenvironments of human vascular systems and analyzing their synergistic effects on the tumor extravasation. Under different mechanical conditions of the vascular system, the tumor cells (HeLa cells) had the highest viability and adhesion activity in the microenvironment of the capillary. The integrity of endothelial cells (ECs) monolayer was destroyed by tumor necrosis factor-α (TNF-α) in a hemodynamic background, which facilitated the tumor cell adhesion, this situation was recovered by the administration of platinum nanoparticles (Pt-NPs). This model bridges the gap between cell culture and animal experiments and is a promising platform for studying tumor behaviors in the vascular system.

Tumor metastasis, leading to over 90% of all tumor-related deaths, is a complex, multistep process including growth, local invasion, intravasation, circulation in blood/lymphatic system, extravasation, and eventually form metastases in remote organs/tissues^1^. Significantly, intravasation of tumor cells into blood vessel and subsequent extravasation of the tumor cells to remote tissues are rate-limiting steps determining tumor metastasis^2^. Numerous studies in the past decade have demonstrated that the tumor cells circulating in vascular system may be used as a biomarker to predict disease progression and survival of cancer patents and guide therapeutic strategy^3^,^4^, therefore, the states of tumor cells in the circulating system and the mechanism of their transferring to potential metastatic sites are of obvious interesting.

Microfluidic cell culture and analysis technologies have enormous potential in building *in vitro* research models that can closely mimic *in vivo* microenvironments^5^,^6^,^7^,^8^,^9^, among which tumor metastasis in circulating system is one of the central issues. The adhesion of tumor cells on endothelial layers in the process of intravasation^10^ and the trans-endothelial invasion of tumor aggregates in the process of extravasation^11^ were investigated on microfluidic chips. Studies carried out on three-dimensional microfluidic models revealed that endothelial barrier impairment was associated with intravasation and extravasation of tumor cells in vascular system^12^, and the presence of tumor cells increases endothelial permeability^13^. These studies greatly enhanced our understanding of mechanisms underlying tumor metastasis. However, during the intravasation, circulation in vascular system, and extravasation, tumor cells must undergo considerable mechanical stimulations including deformations of tumor cells^14^ and hemodynamic forces including fluid shear stress (FSS) and cyclic stretch (CS)^15^. All of these mechanical stimulations could affect survival of the tumor cells and their ability to establish metastatic foci^16^. Although the effects of interstitial flow on morphology^17^ and migration^18^ of tumor cells has been studied, however, little is known about how hemodynamic forces influence viability, proliferation, motion, deformation of tumor cells, and their interplay with endothelial cells (ECs). Therefore, a clear understanding of the role of mechanical environment in the behavior of tumor cells would provide new insights into the metastasis of tumors.

In this study, we systematically investigated the metastatic behavior of model tumor cells (HeLa cells) on a research model based on microfluidic chip. We replicated mechanical environment of vascular system on the chip by applying FSS and CS individually or simultaneously on the tumor cells and ECs. By introducing tumor-related chemical factors in the culture medium, we can also investigate the behaviors of the tumor cells under synergistic effect of mechanical and biochemical microenvironments. On the model, mechanics dramatically influenced the viability of the HeLa cells and the ability of the HeLa cells to adhere on ECs monolayer. The HeLa cells had the lowest apoptosis ratio and the highest probability to adhere on the ECs monolayer in the capillary. In a physiological mechanical background, as a typical biomechanical factor, TNF-α destroyed the integrity of the ECs monolayer and facilitated the HeLa cell adhesion. This model would be an ideal platform for investigation of tumor cell behaviors in the vascular system.

## Results

### Structure and function of the tumor extravasation research model

**Figure 1:**
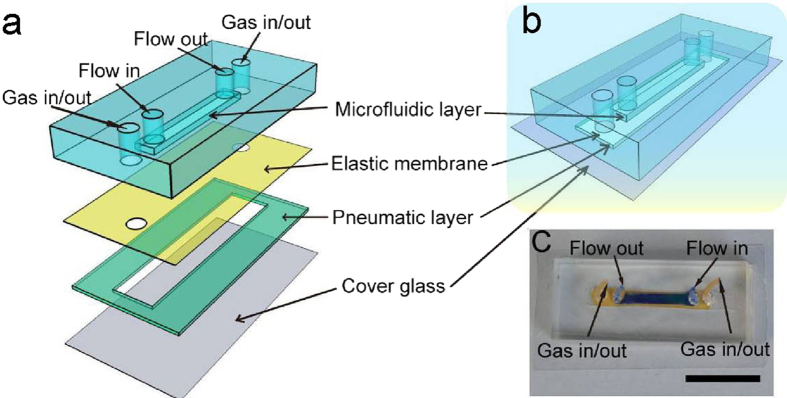
Schematic illustration of the vascular model chip. (**a**) Details of the components of the chip. (**b**) The structure of the integrated chip. (**c**) A photograph of an actual chip. The dark blue part delineates the microfluidic channel and the orange part indicates the stretch chamber. Scale bar: 1.5 cm.

The vascular niche imitating microfluidic chip is composed of four parts: a microfluidic layer, an elastic membrane, a pneumatic layer, and a cover glass (Fig. 1). Apart from the cover glass, other parts of the chip were made of polydimethysiloxane (PDMS). There is a microfluidic channel (height 0.2 mm, width 2 mm, length 20 mm) at the bottom of the microfluidic layer (Fig.1a,b). The elastic membrane (100 μm thick) which is between the microfluidic layer and the pneumatic layer, serves as the bottom of the microfluidic channel. There is a pneumatic chamber (height 0.3 mm, width 0.3 mm, length 30 mm) at the central part of the pneumatic layer. The cover glass (170 μm thick) serves as the bottom of the pneumatic chamber. When vacuum is applied, the elastic membrane and adhered cells will undergo deformation process (Fig. S2a). The entire chip is biocompatible, homogeneous, isotropic and optically transparent (Fig. 1c). So, we can observe and record the whole experimental process by microscopy in real-time. Furthermore, the distance from the cell culture layer to cover glass is less than 270 μm (when the elastic membrane adheres to the cover glass closely) which is within the depth of field of the high resolution lens (Leica, DMI 6000, Germany), the fine structure and the dynamic process of the cells can be directly observed and recorded. We utilized human umbilical vein endothelial cells (HUVEC) as model ECs which can form a monolayer on the elastic membrane to serve as inner layer of the blood vessel.

### The fates of tumor cells in hemodynamic environment

**Figure 2:**
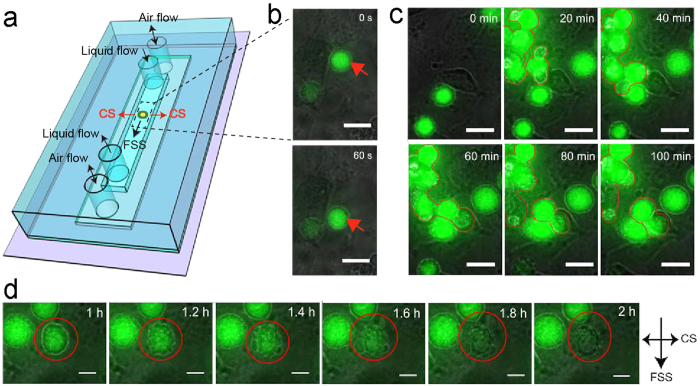
Time-lapse images of HeLa cells under a physiological hemodynamic condition (FSS, 36.2 dynes cm^−2^, CS, 7.2 ± 1.1%, 1.17 Hz). All the cells were stained with Calcein AM. (**a**) The schematic diagram illustrates flow direction of the fluid and positions of the tumor cells in the microchannel. (**b**)The red arrows indicate the travelling trace of a HeLa cell in the microchannel and its attachment on the substrate. (**c**) The process of the formation and breakdown of tumor cell clusters in the microchannel. The red lines track the same cells at different time points. (**d**) The intensity of the fluorescence of the cell highlighted by the red circle experienced an obvious decreasing process. Scale bar: 20 μm. The two-headed arrows indicate the direction of the CS, the one-headed arrows indicate the direction of the FSS.

We studied the behavior of tumor cells in simulated hemodynamic environment. As invaders of the vascular system, tumor cells face complex, harsh environment among which hemodynamic condition plays important role in determining their fates^16^. However, the detail of the interplay between tumor cells and the mechanics in blood vessel is still unclear. Here, by means of the model chip, we systematically studied how tumor cells experience and survive their mechanical microenvironment during their circulating process in the vascular system. We selected HeLa cells as model tumor cells and set physiological hemodynamic condition on the chip (FSS, 36.2 dynes cm^−2^, CS, 7.2 ± 1.1%, 1.17 Hz). The schematic diagram shows the directions of FSS and CS and the relative position of a tumor cell in the microfluidic channel (Fig. 2a). Real-time imaging showed that the tumor cells floating in the medium moved forward in the direction of the fluid flow (Fig. 2b), the cells slowed down their travelling speed and gradually attached onto the substrate (Fig. 2b, Movie S1). Most of the cells separately distributed in the flow at the initial stage (Fig. 2c). With the increase of flowing time, some of the tumor cells aggregated and formed cellular clusters which slowed down their speed and attached to the substrate (Fig. 2c, Movie S2). Previous studies have reported that tumor cell cluster could be found in peripheral blood^19^, here, our study verified the existence of this phenomenon which may be crucial in clogging of tumor cells in the capillaries and facilitating extravasation of the tumor cells. On the chip, some of the tumor cell clusters can also break to separated cells floating in the channel or adhering to the substrate or attaching to other adhered cells (Fig. 2c). We noticed that under the hemodynamic condition, the intensity of the fluorescence of some tumor cells decreased accompanying the gradual disappearance of the edge of the cells at the time point of 1 h (Fig. 2d). At the time point of 2 h, the fluorescence of the cells disappeared completely, leaving only relics of the cells (Fig. 2d). We speculate that the hemodynamic condition may influence the viability of the tumor cells.

**Figure 3:**
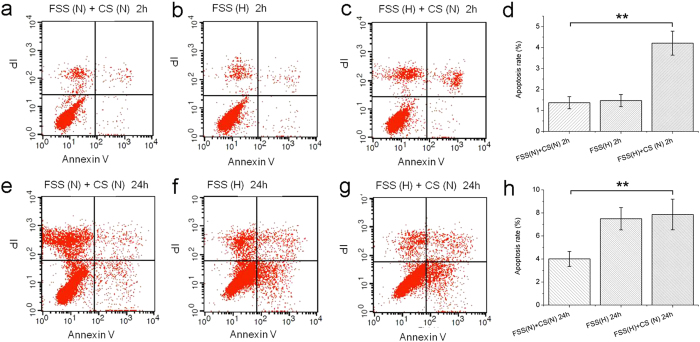
Flow cytometry results indicate the effect of mechanical stimulation on apoptosis of the tumor cells. The red dots in lower right quadrant of each flow cytometry diagram indicate apoptotic cells. (**a**) The apoptosis rate of tumor cells under physiological FSS and CS for 2 h. (**b**) The apoptosis rate of tumor cells under high level of FSS for 2 h. (**c**) The apoptosis rate of tumor cells under high level of FSS and physiological CS for 2 h. (**d**) The statistical data of the tumor apoptosis rates under different conditions for 2 h. (**e**) The apoptosis rate of tumor cells under physiological FSS and CS for 24 h. (**f**) The apoptosis rate of tumor cells under high level of FSS for 24 h. (**g**) The apoptosis rate of tumor cells under high level of FSS and physiological CS for 24 h. (**h**) The statistical data of the tumor apoptosis rates under different conditions for 24 h. **Conditions that were statistically different (P < 0.01). All results are shown plotted as mean ± SD with each group containing three experiments.

To explore whether the disappearance of the fluorescence of the tumor cell in Fig. 2d indicates its apoptosis, the apoptosis assay of tumor cells in different hemodynamic conditions was carried out on flow cytometry. Under a physiological mechanical condition (FSS, 36.2 dynes cm^−2^, CS, 7.2 ± 1.1%, 1.17 Hz) for 2 h, apoptosis took place in about 1.37 ± 0.29% of the tumor cells (Fig. 3a,d). When the mechanical condition changed to a high level FSS (76.9 dynes cm^−2^, no CS) for 2 h, the apoptosis rate of the tumor cells increased to 1.47 ± 0.3% (Fig. 3b,d). Under the high level FSS and a physiological CS (FSS, 76.9 dynes cm^−2^, CS, 7.2 ± 1.1%, 1.17 Hz), the apoptosis rate of the tumor cells rose to 4.21 ± 0.57% during the same stimulation period (Fig. 3c,d), which was significantly higher than that of the physiological mechanical condition (Two-sample *t*-test, *P* < 0.01, Fig. 3d). These results indicate that the harsher the mechanical condition, the higher apoptosis rate of the tumor cells. In human body, tumor cells may circulate in the vascular system for a long time, so, we prolonged the observation time under the same mechanical stimulations. Under the physiological condition (FSS, 36.2 dynes cm^−2^, CS, 7.2 ± 1.1%, 1.17 Hz), the high level FSS (76.9 dynes cm^−2^, no CS), or the high level FSS plus the physiological CS (FSS, 76.9 dynes cm^−2^, CS, 7.2 ± 1.1%, 1.17 Hz) for 24 h, the apoptosis rates of the tumor cells were 4.01 ± 0.65%, 7.49 ± 0.97%, and 7.86 ± 1.33% respectively (Fig. 3e–h). Under the high FSS, the apoptosis rate were significantly higher than that under physiological mechanical condition (Two-sample *t*-test, *P* < 0.01, Fig. 3h). By comparison, the apoptosis rate of the tumor cells stimulated for 24 h were significantly higher than those under the same stimulations for 2 h (Fig. 3d,h). Thus, the extent of the apoptosis of the tumor cells was time-dependent. Tumor cells are not original blood cells, therefore the environment in vascular system is harsh for them. Our study provides the direct evidence that mechanics in blood stream can influence the survival of the tumor cells.

### Tumor cell-EC monolayer interactions in hemodynamic environment

After travelling in peripheral blood circulation, the surviving tumor cells must adhere to the vascular endothelium to start the extravasation process^16^. It is known that mechanical environment plays key role in influencing tumor cell adhesion and subsequent migration steps^16^. However, the precise mechanism of the adhesion of tumor cells on EC monolayer remains elusive, the lack of appropriate research model is a major reason for this situation. In this study, the individual or combined FSS and/or CS stimulations were applied to HeLa cells to investigate the detailed process of tumor cell adhesion in different hemodynamic environments.

**Figure 4:**
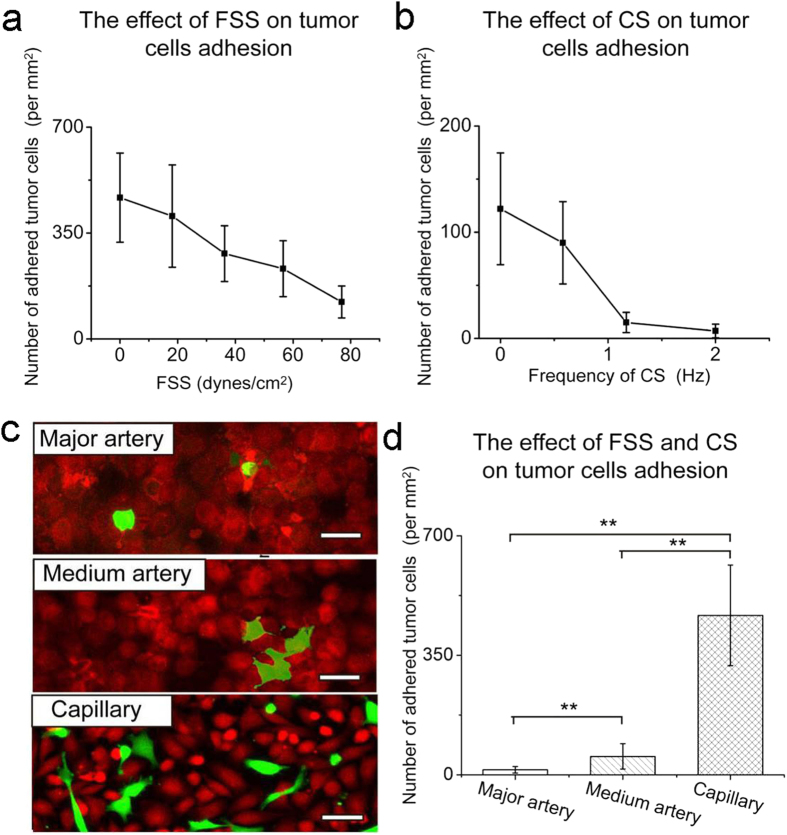
The single or combined impact of FSS and CS on the adhesion of the tumor cells on the chip. (**a**) The effect of different levels of FSS on the adhesion of the tumor cells on the ECs monolayer. (**b**) The effect of different levels of CS on the adhesion of the tumor cells on the ECs monolayer. (**c**)The adhesion of the tumor cells (HeLa, green) on the ECs monolayer (HUVEC, orange) under different mechanical conditions simulating microenvironments of main artery, medium-sized artery, and capillary respectively for 2 h. Scale bar, 10 μm. (**d**) The statistical result of (**c**). **Conditions that were statistically different (P < 0.01).

The effect of FSS intensity on the tumor cell adhesion was evaluated. According to the physiological FSS range in human vascular system (15–70 dynes cm^−2^), we selected four levels of intensity of FSS (18.1, 36.2, 56.6, 76.9 dynes cm^−2^) in this experiment, setting static condition as a control. Confluent EC monolayer formed on the substrate of the microchannel before the introduction of tumor cells. In the static condition, some of the tumor cells gradually attached and adhered on the EC monolayer (average 467 per mm^2^), whereas other cells kept suspending in the culture medium over the observation time (2 h) (Fig. 4a). At the FSS intensity of 18.1 dynes cm^−2^, the tumor cells initially floated over the EC monolayer at a suspending state. Afterwards, some tumor cells began to roll on the EC monolayer, slowed down and finally stopped rolling within several minutes. The number of adhered cells (average 406 per mm^2^) decreased apparently compared with the static condition (Fig.4a). The rest of the tumor cells did not adhere during the whole observation time (2 h). With the increase of FSS intensity from 18.1 to 76.9 dynes cm^−2^, within the same observation time (2 h), the adhesion ratio of the HeLa cells decreased dramatically (36.2 dynes cm^−2^, ∼282 per mm^2^; 56.6 dynes cm^−2^, ∼232 per mm^2^;76.9 dynes cm^−2^, ∼122 per mm^2^)(Fig. 4a). The number of adhered cells at 76.9 dynes cm^−2^ was about one fourth of that at static condition (Fig. 4a), demonstrating that FSS is a key factor influencing the adhesive properties of the tumor cells.

We next evaluated the effect of CS on tumor cell adhesion on EC monolayer, since CS is another key mechanic factor existing in blood vessel, especially in the artery. In terms of the physiological heart beat rates of human, we tested the adhesive properties of HeLa cells on the chip under four levels of CS: 0 Hz (static condition), 0.6 Hz (bradycardia), 1.17 Hz (physiological), and 2 Hz (tachycardia) respectively. Under 2 h of static condition, average 122 per mm^2^ of the tumor cells adhered on the EC monolayer (Fig. 4b); when the CS frequency changed to a bradycardiac condition, there were about 90 per mm^2^ tumor cells adhered on the EC monolayer (Fig. 4b); when the tumor cells stayed in a circumstance with physiological heart rate, the number of adhered cells decreased dramatically (15 per mm^2^) (Fig. 4b). Under a tachycardiac condition, the number of adhered tumor cells was the lowest (7 per mm^2^) among the four levels of CS conditions (Fig. 4b). So, the number of adhered tumor cells on EC monolayer was inversely related to the CS frequency (Fig. 4b).

In the vascular system, heart and artery, especially large artery such as aorta, can scarcely be invaded by tumor cells to form metastatic tumor^16^, by contrast, tumor metastasis usually takes place in blood supply-rich tissues or organs such as lung, liver, and bone where tumor cells slow down their traveling speeds in small arteries and ultimately adhere to the wall of capillary. How hemodynamic factors influence the adhesion of tumor cells at different sites of vascular system is significant for both fundamental and clinical research in tumor metastasis. In this study, we simulated hemodynamic environments of major artery, medium-sized artery, and capillary separately by setting different stimulation conditions. We set 76.9 dynes cm^−2^ of FSS and 1.17 Hz of CS to simulate major artery where FSS and CS are both dominating mechanical forces exerting on ECs and thus tumor cell-EC interactions. As shown in Fig. 4c, only few tumor cells adhered on the EC monolayer during 2 h of observation. We also simulated tumor cells in medium-sized artery and capillary by setting corresponding parameters (medium-sized artery, 36.2 dynes cm^−2^ of FSS and 1.17 Hz of CS; capillary, 0 dynes cm^−2^ of FSS and 0 Hz of CS). The number of the adhered tumor cells increased significantly with the decrease of FSS intensity (major artery & medium-sized artery, *P* < 0.01, Two-Sample *t*-test) (Fig. 4c,d). Apparently, the absence of CS and the lowered intensity of FSS led to the increase of the adhered tumor cells on the EC monolayer (major artery & capillary, *P* < 0.01, Two-Sample *t*-test; medium-sized artery & capillary, *P* < 0.01, Two-Sample *t*-test) (Fig. 4c,d). Thus both FSS and CS impeded the adhesion of the tumor cells to the EC monolayer. The result is well in accord with *in vivo* situations where tumor cells rarely metastasis through large artery, while organs such as liver and lung with plenty of capillaries are usually targets of secondary tumors mainly come from tumor cells circulating in blood.

### Tumor-EC monolayer interaction in TNF-α conditioned biochemical environment

**Figure 5:**
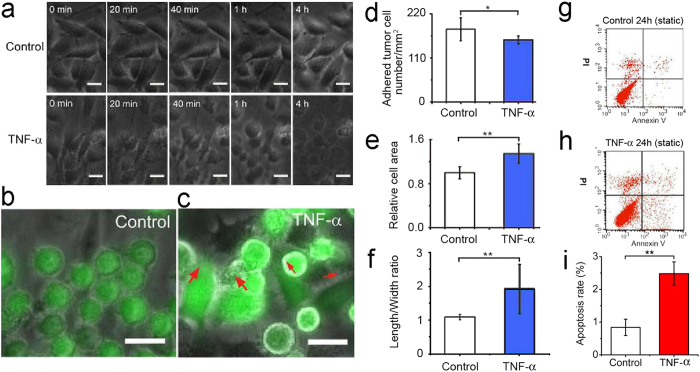
Tumor cells-ECs monolayer interaction in TNF-α conditioned biochemical environment. (**a**) The morphological change of the ECs monolayer in the absence or in the presence of TNF-α. (**b**) The adhesion of the tumor cells on the ECs monolayer and their morphological changes in the absence of TNF-α. (**c**) The adhesion of the tumor cells on the ECs monolayer and their morphological changes in the presence of TNF-α. In (**b,c**), the tumor cells were stained with Calcein Green AM. Scale bar, 20 μm. (**d**) The number of adhered tumor cells on the ECs monolayer in the absence or presence of TNF-α. (**e**) The spreading area of adhered tumor cells on the ECs monolayer in the absence or presence of TNF-α. (**f**) The aspect ratio of the adhered tumor cells in the absence or presence of TNF-α. (**g**) The result of flow cytometry indicates the apoptosis rate of the tumor cells in the absence of TNF-α at static condition for 24 h. (**h**) The result of flow cytometry indicates the apoptosis rate of the tumor cells in the presence of TNF-α at static condition for 24 h. (**i**) Statistical data of the tumor cell apoptosis in the absence or presence of TNF-α at static condition for 24 h. *Conditions that are statistically different (*P* < 0.05). **Conditions that were statistically different (P < 0.01). All results are shown plotted as mean ± SD with each group containing three experiments. Scale bar, 20 μm.

TNF-α is one of the important inflammatory cytokines that can promote cancer metastasis^20^. We evaluated the role of TNF-α on the behavior of HeLa cells in the model vascular system. In medium without TNF-α, the EC monolayer maintained the intact state within 4 h (Fig. 5a). However, when the ECs were cultured in TNF-α conditioned medium (75 ng mL^−1^), they contracted. This tendency became more apparent with the increase of culturing time (Fig. 5a). Finally the cells detached from the substrate and only left residues, indicating that TNF-α can destroy the integrity of the EC monolayers and lead to the dysfunction or even death of the ECs.

The HeLa cells in medium without TNF-α can maintain separated states and attach to the surface of the EC monolayers within 2 h in a static situation (Fig. 5b). By contrast, within 2 h, the HeLa cells in the medium containing TNF-α (75 ng mL^−1^) aggregated and adhered on the surface of the EC monolayer firmly followed by the spread of the adhered HeLa cells and their infiltration into the space of the adjacent ECs (Fig. 5c). By measuring the number of the cells, we found that the presence of TNF-α led to the decrease of the number of suspending HeLa cells in the medium and subsequently the lower number of adhered tumor cells on the EC monolayer, showing that TNF-α can affect the viability of the HeLa cells (Fig. 5d) Two sample *t*-test, *P* < 0.05). The measurement of the spreading area of the adhered HeLa cells indicates that the HeLa cells adhered on the EC monolayer in TNF-α conditioned medium had larger spread areas than those in medium without TNF-α (Fig. 5e, Two sample *t*-test, *P* < 0.01), this means that the TNF-α-treated HeLa cells had more contact areas with the substrate than the untreated cells. Furthermore, we measured the aspect ratio of the HeLa cells and found that the TNF-α-treated cells had larger aspect ratios than those of the untreated cells (Fig. 5fTwo sample *t*-test, *P* < 0.01), demonstrating that TNF-α can promote the interaction between the HeLa cells and the ECs to strengthen the invasive property of the HeLa cells. Our study is consistent to recent report which indicated that TNF-α can weaken the integrity of ECs monolayer and promote the penetration of tumor cells through the ECs monolayer^12^.

To verify the effect of TNF-α on tumor cell viability, HeLa cells were incubated in culture medium with or without TNF-α (75 ng mL^−1^) for 24 h at static condition and characterized by flow cytometry. Apoptosis took place in 0.84 ± 0.25% of the HeLa cells cultured in the medium without TNF-α within 24 h (Fig. 5g,i). By contrast, the apoptosis ratio rose to 2.48 ± 0.36% for the tumor cells in medium containing TNF-α (Fig. 5h,i), which is significantly higher than that at TNF-α-free medium (Fig. 5i, Two sample *t*-test, *P* < 0.01), implying that TNF-α can decrease the viability of the tumor cells.

### Tumor cells in whole blood on the chip

**Figure 6:**
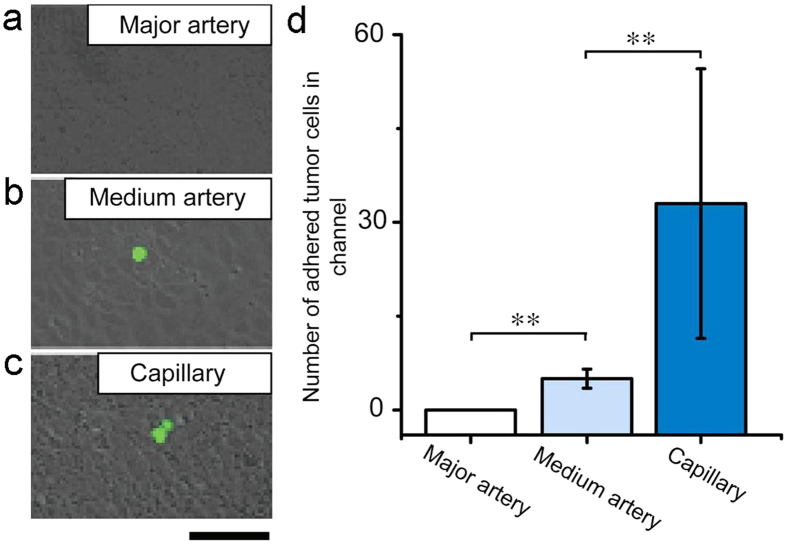
HeLa cells in whole blood on the chip. (**a**) The adhesion of HeLa cells on the wall of the simulated major artery. (**b**) The adhesion of HeLa cells on the wall of the simulated medium-sized artery. (**c**) The adhesion of HeLa cells on the wall of the simulated capillary. (**d**) The statistic result of the numbers of adhered HeLa cells on the walls of different simulated blood vessels. **Conditions that are statistically different (*P* < 0.01). All results are shown plotted as mean ± SD with each group containing three experiments. Scale bar, 100 μm.

We further recapitulated vascular environments for the tumor cells and ECs on the chip with real human blood. The blood mixed with small amount of HeLa cells was introduced into the ECs-pre-coated microchannel. Under a hemodynamic condition of major artery (FSS, 76.9 dynes cm^−2^, CS, 1.17 Hz), no HeLa cell adhered on the EC monolayer (Fig. 6a). Under a hemodynamic condition of medium-sized artery (FSS, 36.2 dynes cm^−2^, CS, 1.17 Hz), only few HeLa cells adhered on the surface of EC layer (Fig. 6b), being significantly different from the condition of major artery (*P* < 0.01, Two-Sample *t*-test) (Fig. 6d). When the hemodynamic condition was changed to that in capillary (FSS, 0 dynes cm^−2^, CS, 0 Hz), several HeLa cells adhered on the EC layer (Fig. 6c). The condition of the capillary significantly promoted the adhesion of the HeLa cells compared with the condition of medium-sized artery (*P* < 0.01, Two-Sample *t*-test) (Fig. 6d). In summary, in whole blood, the HeLa cells can hardly adhere on the EC monolayer in simulated hemodynamic condition of large blood vessel, they prefer to adhere to the EC monolayer in a capillary-like condition.

### Nanodrug treatment on the model

**Figure 7:**
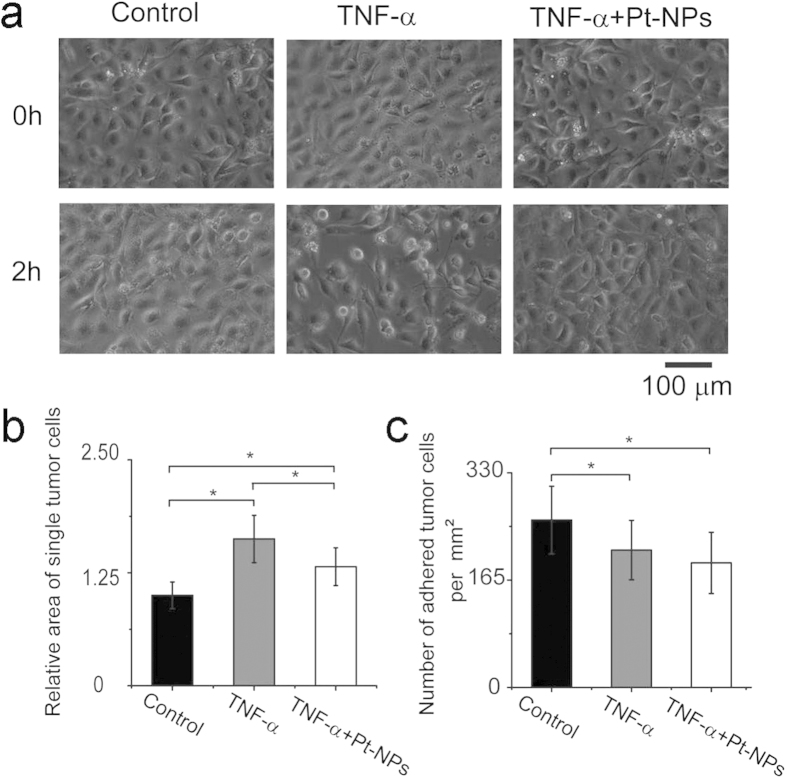
Pt-NPs treatment on the model. (**a**) The morphological changes of the ECs monolayers under control conditions, treated with TNF-α, treated with TNF-α + Pt-NPs, respectively. (**b**) The areas of the tumor cells adhered on the ECs monolayer under three different conditions: control, treated with TNF-α, treated with TNF-α + Pt-NPs, respectively. (**c**) The number of adhered tumor cells on the ECs monolayer under three different conditions: control, treated with TNF-α, treated with TNF-α    + Pt-NPs, respectively. *Conditions that are statistically different (*P* < 0.05). All results are shown plotted as mean ± SD with each group containing three experiments.

The integrity of ECs monolayer plays key roles in the maintenance of vascular homostasis. Reactive oxygen species (ROS), generated by ECs in adverse conditions such as inflammation or aberrant mechanical stimulations, can effectively inhibit the activity of phosphatases that regulate the function of VE-cadherin and weaken the EC-EC connections to facilitate the extravasation of blood cells^21^. Platinum nanoparticles (Pt-NPs) has superoxide dismutase (SOD) catalytic activity and is a good candidate for scavenging ROS in various diseases^22^,^23^. In this study, we evaluated if Pt-NPs can protect the integrity of the ECs monolayer and prevent the invasion of tumor cells. Compared with the control (without TNF-α and/or Pt-NPs), the presence of TNF-α for 2 h led to apparent contraction of the ECs and the compromise of their interconnection (Fig. 7a). The treatment of Pt-NPs effectively prevented the ECs from contraction and the disruption of the cell-cell connections within 2 h (Fig. 7a). The spreading area of the tumor cells adhered on the ECs monolayer in TNF-α conditioned medium was larger than that of the cells in control condition (*P* < 0.05, Two-Sample *t*-test) (Fig. 7b). The presence of Pt-NPs evidently decreased the area of the tumor cells adhered on the ECs monolayer (*P* < 0.05, Two-Sample *t*-test) (Fig. 7b). The number of the adhered tumor cells on the ECs monolayer decreased in the presence of TNF-α compared with the control (*P* < 0.05, Two-Sample *t*-test) (Fig. 7c). The treatment of the cells with both TNF-α and Pt-NPs led to further decrease of the number of adhered tumor cells on the ECs, demonstrating that the Pt-NPs evidently hindered the adhesion of the tumor cells to the ECs monolayer and the spread of the tumor cells on the substrate (*P* < 0.05, Two-Sample *t*-test) (Fig. 7c). So, on our model, the Pt-NPs effectively protected the ECs monolayer from suffering the penetration of the tumor cells.

## Discussion

In this study, we constructed a microfluidic vascular model that is capable of simultaneously mimicking both mechanical and biochemical microenvironment of the vascular system. We studied individual or synergistic effects of mechanical and biochemical factors on the behavior of tumor cells, especially their adhesion, on the model. The circulating of tumor cells in vascular system and subsequent extravasation are key steps for tumor metastasis, however, the mechanism underlying tumor cell behaviors in blood stream is still largely unknown. The major reason for this situation is the lack of adequate research tool for real-time observation and studying of dynamic variation of the cellular characteristics. The hydrodynamic microenvironment of vascular system is complex, and essential in tumor extravasation. FSS and CS are two major mechanics in the vascular system. In the previous studies, only the effect of FSS on tumor cells has been addressed^24^. Furthermore, these experiments were carried out on flow chamber or capillary which are incapable of simulating complex flow patterns in real blood vessel^25^. Apart from FSS, another essential mechanical stimulation, CS, has not been addressed in tumor metastasis researches. In this paper, by integrating both FSS and CS on a chip, we realized omnibearing simulating of hydrodynamic condition of vascular system and provided a useful tool for tumor ecology study. Two phenomena, apoptosis and decreased adhesion of the tumor cells under high intensity of mechanical stimulation were observed in our study. Recently, accumulating evidence indicated that hypertension induced by vascular endothelial growth factor (VEGF) inhibitor is closely related to survival ratio of the cancer patients^26^,^27^. One direct effect of hypertension is the harsher mechanical environment for the vascular cells^28^. So, the harsh hemodynamic condition may influence the fates of tumor cells. In recent study, Mitchell *et al.* reported that TNF apoptosis-inducing ligand (TRAIL)-induced tumor cell apoptosis increased in a fluid shear stress force- and time-dependent manner^24^. Our results not only is consistent with previous studies that mechanics can promote apoptotic agents-induced apoptosis, but also demonstrate that the hemodynamic condition alone is capable of inducing tumor cell apoptosis. Our results on the chip demonstrate that mechanical condition in the vascular system contributes to death of tumor cells. This may be helpful for explaining why tumor cells are rare cells in the blood of cancer patients. It also implies that physical activities which can promote blood circulation may be helpful for decreasing the incidence of tumor metastasis.

We also studied simulating more complex conditions on the chip by simultaneously exerting both mechanical (FSS and CS) and biochemical (TNF-α) stimulations on the tumor cells-ECs interaction. On one hand, TNF-α weakened the integrity of the ECs monolayer (Fig. 5a), demonstrating the toxicity of this cytokine on the barrier cells. Thus, TNF-α has the potential to promote tumor cell extravasation. On the other hand, TNF-α promoted tumor cell adhesion on the ECs monolayer (Fig. 5c–f) and exhibited tumor invasion-promoting behaviors. We also found that besides the positive role of TNF-α in tumor cell invasion, this cytokine led to the apoptosis of the tumor cells (Fig. 5h), which is consistent with previous reports^29^. Comparatively, TNF-α led to less apoptosis of tumor cells (2.48 ± 0.36%, Fig. 5h,i) than mechanic stimulations (4.01 ± 0.65%, Fig. 3e,h), demonstrating the significant role of mechanical environment in influencing tumor cell viability.

In our study, under TNF-α treatment, the number of the adhered tumor cells on the ECs monolayer decreased (Fig. 5d), which seemed to be contradictory regarding the enhanced metastatic behavior of the tumor cells. We propose that the result was determined by the balance between the states of the invader (tumor cells) and the barrier (ECs monolayer). Without the disturbing effects of TNF-α, the ECs monolayer can maintain its integrity and hinder the adhesion of the tumor cells. In the presence of TNF-α, although the viability of the tumor cells was compromised, most of them were still alive (more than 90%, see Fig. 5h). On the contrary, most of the inter-cell connections of the ECs were destroyed by the TNF-α treatment. So, the tumor cells had more opportunities to adhere to the spaces between the ECs. Therefore, our results demonstrate that the integrity of the ECs monolayer may be more important than the aggressiveness of the tumor cells during the extravasation process.

In this study, the Pt-NPs effectively recovered the integrity of the ECs monolayer and inhibited the tumor cell adhesion on the ECs monolayer (Fig. 7). According to the previous reports, ROS and cellular oxidant stress are closely associated with cancer^30^. On one hand, ROS can attack DNA and contribute to the induction of mutations of normal cells and the transformation of normal cells to cancer cells^31^. On the other hand, ROS can mediate cell-signaling pathways that are involved in cell growth regulatory pathways. Because DNA damage, mutations and altered gene expression are all key players in the process of carcinogenesis, thus, ROS are instrumental in the process of carcinogenesis, and antioxidant treatment may be beneficial for inhibition of cancer progression. It was reported that cancer cells appear to generate more ROS than their normal counterparts, thus cancer cells should be more tolerant of ROS than for normal cells^32^. TNF-α can induce ROS production and accumulation in cells^33^. In our system, TNF-α was utilized to treat both tumor cells and ECs, which can lead to ROS production in the cells. Subsequently, we explored if antioxidants can hinder the tumor cell-EC monolayer interaction on the chip.

Antioxidants have been reported to play protective role in cancer patents. Vitamin C can reduce the incidence of the stomach, lung, and colorectal cancers^34^. The intake of vitamin E reduced the incidence of colorectal cancer by triggering apoptosis of cancer cells^35^. Pt-NPs have been reported to be effective antioxidants to scavenge ROS for preventing and treating diseases such as pulmonary inflammation^36^ and atherosclerosis^23^. In this study, Pt-NPs effectively recovered the TNF-α-induced break of the ECs monolayer and decreased the number of adhered tumor cells and the spreading area of the adhered tumor cells (Fig. 7). Our study provides the direct evidence that the antioxidant is benefit for maintaining the integrity of the ECs monolayer and suppress the adhesion of tumor cells, the mechanism underlying this process will be addressed in the future research on the chip. The protective role of Pt-NPs in preventing tumor cell adhesion on the ECs monolayer further demonstrates the robust of this nanodrug not only in ROS dominated diseases, but also in cancers, implying an alternative way to reduce the incidence of cancer both for individual and population by antioxidant administration.

## Methods

### Fabrication of the microfluidic chip

Both the microfluidic layer and the pneumatic layer were produced by replica molding. The elastic membrane was generated by spin-coating PDMS pre-polymer (10: 1, base to curing agent) on a smooth silanized wafer. A 100 μm-thick PDMS membrane was produced by spin-coating at 1000 rpm for 60 s and subsequent curing at 70 °C for 2 h. All the parts of the chip were irreversibly bonded together by using oxygen plasma treatment (Harric plasma cleaner, Ithaca, NY, USA).

### Simulating the vascular microenvironment on the chip

The mechanical environment in human vascular system is extremely heterogeneous, ranging from pulsatile flow in major and medium-sized arteries to slow or intermittent flow in capillaries and veins^37^. To mimic the vascular mechanical environment, the microfluidic chip was connected to a flow driving system and a vacuum generating system. Fig. S1 shows the schematic of the components of the whole system which can provide individual or combined FSS and CS to the cells on the chip. The inlet and outlet of the microfluidic channel were connected to a flow-driving system including a peristaltic pump and a culture medium reservoir to generate FSS on the cells. The physiological range of FSS on vascular walls varies within 15–70 dynes cm^−2^ over the cardiac cycle^38^. We selected four different magnitudes of FSS: 0 dynes cm^−2^, 18.1 dynes cm^−2^, 36.2 dynes cm^−2^, and 76.9 dynes cm^−2^ as no, low, physiological, and high FSS stimulation for the cells. The FSS on vascular walls was calculated by the equation (1)^39^:


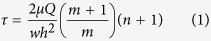


Where *τ* is the wall shear stress, *μ* is the viscosity of the medium, *Q* is the flow rate of liquid, *w* and *h* are the width and the height of the channel, respectively. *m* and *n* are empirical parameters.

The stretch chamber was connected to a vacuum generation system (LianYing Tech. Co., Ltd, Beijing, China). The system can generate various cyclic airflows and subsequent different CS on the chip. We set the frequency of stretch at 0.58 Hz, 1.17 Hz, and 2 Hz to mimic low, physiological, and high heart rate to investigate the effect of CS on the cells.

To mimic hemodynamic conditions in different parts of the circulating system, we set three kinds of simulating conditions: 1) Major artery, FSS (76.9 dynes cm^−2^) plus CS (7.2 ± 1.1%, 1.17 Hz); 2) Medium-sized artery, FSS (36.2 dynes cm^−2^) plus CS (7.2 ± 1.1%, 1.17 Hz); 3) Capillary, no FSS (0 dynes cm^−2^) plus CS (0%, 0 Hz). The real-time behavior of the cells on the chip can be imaged by microscopy with high-resolution lens.

### Chip characterization

The homogeneity and repeatability of the elastic membrane were characterized by stretching adhered HeLa cells stained by Calcein AM (3.5 μg mL^−1^, Invitrogen) and measuring the variation of the distances between the cells. Fig. S2A shows the schematic of the stretching process. When vacuum is applied, the elastic membrane bends down and attaches to the surface of the cover glass which serves as the bottom of the microfluidic channel. The attached part of the membrane becomes flat whereas the two side parts of the membrane are suspended and arc-shaped. Only the flat part of the membrane is suitable for observing under microscopy. We divide the flat part of the membrane into three areas: one central area (width, 1200 μm) and two side areas (width, 800 μm) (Fig. S2A). The stretch ratio of the central area is 7.2 ± 1.1% and two side areas are 8.46 ± 1.48%, respectively (Fig. S2B, S2C). Due to the uneven distribution of the stretching in the three areas of the membrane (*P* < 0.05, Two-Sample *t*-test), we only observe and analyze cells in the central area of the membrane to ensure the consistency of the results.

We tested the high resolution imaging ability of the chip by observing cells exposed to mechanical stimulations. Briefly, HUVEC were cultured in the channel for 2 h to adhere, then single or combined FSS and CS stimulations were applied to the cells. The dynamic images of the HUVECs stained by Tubulin Tracker Green (Molecular Probes) were recorded by a confocal microscope (LSM700, Carl Zeiss). Fig. S3A shows the dynamic change of the cells under FSS (36.2 dynes cm^−2^). The black arrow indicates the direction of FSS. Red circles in the series of images highlight the disassembly and reassembly processes of tubulins in the cells. Fig. S3B shows the dynamic imaging of HUVECs under CS (7.2 ± 1.1%, 1.17 Hz). The pink lines with two arrow heads in the images indicate the variation of the width of a cell at stretched and relaxed states at different time points. We also characterized the dynamic states of HUVECs under synergistic effects of FSS (36.2 dynes cm^−2^) and CS (7.2 ± 1.1%, 1.17 Hz) (Fig. S3C). The pink lines with two arrow heads indicate the variation of the cell size during the FSS and CS stimulations.

### Cell loading and culture on the chip

Both HUVECs and HeLa cells were provided by Cell Culture Center of Chinese Academy of Medical Sciences. The cells were cultured in DMEM culture medium (Gibco) containing 10% (v/v) fetal bovine serum (FBS) and 1% (v/v) penicillin/streptomycin. Before cell loading, the chip, medium reservoir, and tubings were sterilized with 75% ethanol and thoroughly washed with phosphate-buffered saline (PBS) twice. To enhance cell adhesion, fibronectin solution (10 μg mL^−1^ in PBS) was introduced into the flow channel and incubated at 37 °C for 30 min. The channel was washed with PBS to remove non-adhered fibronectin. HUVECs in Petri dishes were washed with PBS and trypsinized with 0.05% trypsin in 1 mM ethylenediaminetetraa-cetic acid (EDTA, Gibco). The cells were suspended in cell culture medium with a density of about 10^5^ cells mL^−1^. The cell suspension was introduced into the flow channel by a microsyringe. After culturing in the cell incubator (37 °C, 5% CO_2_) for 2 h, the chip was connected to the flow-driving system (18.1 dynes cm^−2^, within physiological FSS level and low enough to avoid violent stimulations to the cells) to provide the cells a fresh growing environment. HUVECs in the channel were cultured (37 °C, 5% CO_2_) for 24 h to form a confluent ECs monolayer for experiments. HeLa cells were used as model tumor cells in this study. HeLa cells were introduced into the flow-channel at a concentration of 3 × 10^4^ mL^−1^. The concentration of the HeLa cells in this study was much higher than that in real patient’s blood. There are two reasons for this: one is to increase the probability of interaction between tumor cells and HUVECs; the other is to imitate the collision among cells in blood stream since the collision process in the peripheral blood circulation is also an important mechanical factor influencing the fates of tumor cells. By elevating cell concentration, we can conveniently investigate cell-cell interactions, the rare events *in vivo*, on a chip conveniently.

### Live cell staining and imaging on the chip

In this study, Calcein Green AM (3.5 μg mL^−1^, Invitrogen) and Calcein Orange AM (5 μg mL^−1^, Invitrogen) were used to stain live cells. The staining was performed by slowly introducing the dye solutions into the microfluidic channel via tubing and incubating in incubator (37 °C and 5% CO_2_) for 45 min. Then the excess dyes were washed away by cell culture medium. All the staining processes were performed in darkness. Tubulin Tracker Green (Molecular Probes) staining was used to facilitate live cell imaging. Briefly, cells adhered on the chip were rinsed with PBS followed by treating with 250 nM Tubulin Tracker Green in culture medium mixed with 10% F127 (Molecular Probes) for 30 min in an incubator (37 °C, 5% CO_2_). The cells were washed with PBS before observation. A confocal microscope (LSM700, Carl Zeiss) was used to observe and record the behavior of the cells in real time.

### Pt-nanoparticles (Pt-NPs) synthesis

Pt-NPs (average diameter 2 nm) were synthesized by a modified ethanol reduction method^40^. 0.04% hydrogen hexachloroplatinate, 40 mM sodium phosphate buffer (pH 7.0), 0.5% polyethylene glycol sorbitan monooleate (Tween 80) and 10% ethanol was incubated in a sealed bottle at 60 °C until the mixture became black. The solution of Pt-NPs was desalted with Milli-Q water (1:10) and dialysed with a 10000 molecular weight limiting dialysis membrane. Above two steps were repeated three times. The concentration of Pt-NPs was tested by inductively coupled plasma mass spectrometry (ICP-MS, Agilent 7500, Agilent Technologies, CA). The Pt-NPs solution was stored at 4 °C until use.

### Chemical stimulations

TNF-α, an important biochemical factor in cancer metastasis, was introduced on the chip to evaluate its effects on tumor cells and their interactions with ECs^41^. HUVECs were cultured on the chip for 24 h under FSS (36.2 dynes cm^−2^) before the experiment. After the addition of TNF-α (75 ng mL^−1^, Peprotech), the cells were cultured (37 °C, 5% CO_2_) for 4 h under the same mechanical stimulation. The effect of TNF-α on tumor cells was investigated by culturing HeLa cells in TNF-α (75 ng mL^−1^)-conditioned medium for 24 h at static condition. The tumor cells-HUVECs interaction was conducted in TNF-α conditioned medium (75 ng mL^−1^) in the hemodynamic environment (FSS, 36.2 dynes cm^−2^ CS, 7.2 ± 1.1%, 1.17 Hz).

### Cell apoptosis assay

We analyzed the apoptosis of the cells by a flow cytometer (BD FACS Calibur). Briefly, HeLa cells circulated in the microfluidic system and interacted with ECs monolayer under the condition of FSS and/or CS for 2 h or 24 h respectively. After the stimulation on the chip, the non-adhered HeLa cells were immediately collected with the same flow as the experimental condition and stained by a Annexin V Apoptosis Detection Kit (eBioscience) following the instruction. Firstly, cells were rinsed with PBS and the binding buffer, and resuspended in the binding buffer at a concentration of 1 × 10^6^ mL^−1^; 5 μL of fluorochrome-conjugated Annexin V was added into 100 μL of the cell suspension and incubated for 15 min at ambient temperature; the cells were rinsed and resuspended in 200 μL of the binding buffer. 5 μL of Propidium Iodide staining solution (cat. 00–6990, eBioscience) was added into the cell suspension to indicate the necrosis of the cells. The samples were kept at 4 °C in darkness until use. All the independent experiments by FACS were repeated at least 3 times.

### Tumor cell adhesion experiment

We studied the effect of FSS, CS, and TNF-α (75 ng mL^−1^) on the adhesion of tumor cells to ECs monolayer on the chip. Briefly, the HeLa cell suspension was driven by a peristaltic pump at different flow rates to travel through microfluidic channels and interact with pre-formed ECs monolayer for 2 h. The real-time adhesion process of the HeLa cells on the ECs monolayer was recorded by optical microscopy (Movie S1 and S2). After the FSS, CS and TNF-α stimulations, non-adherent HeLa cells were washed out of the channel by the same flow as the experimental condition, before the number of adhered HeLa cells was counted and compared for analysis. To evaluate cellular response to TNF-α stimulation, we measured and analyzed the variation of the area of the tumor cells to characterize their states. We also measured the aspect ratio of the cells to evaluate the adhesive property of the cells. Aspect ratio = Length of the cell/Width of the cell. Aspect ratio ≥ 1. If the aspect ratio approaches 1, it indicates that the cell is nearly round-shaped and is poorly adhered. The larger the aspect ratio, the more active the cell to adhere, spread, and migrate.

### Statistics and analysis

In this study, all the experiments were repeated at least 3 times and the results were reported as mean ± standard error. The comparisons between two groups were analyzed by using Two-sample *t*-test.

## Additional Information

**How to cite this article**: Huang, R. *et al.* Investigation of Tumor Cell Behaviors on a Vascular Microenvironment-Mimicking Microfluidic Chip. *Sci. Rep.* **5**, 17768; doi: 10.1038/srep17768 (2015).

## Supplementary Material

Supplementary Information

Supplementary Movie S1

Supplementary Movie S2
