# Supplementary material for: Investigation of Tumor Cell Behaviors on a Vascular Microenvironment-Mimicking Microfluidic Chip
Source: Sci Rep. 2015 Dec 3;5:17768. doi: 10.1038/srep17768 (PMC4668571; doi:10.1038/srep17768)
Supplement: Supplementary Information [file srep17768-s1.doc]

**Supporting Information**

**Investigation of Tumor Cell Behaviors on a Vascular Microenvironment-Mimicking Microfluidic Chip**

Rong Huang^1^, Wenfu Zheng^2^*, Wenwen Liu^2^, Wei Zhang^2^, Yunze Long^1^*, Xingyu Jiang^2^*

^1^College of Physics & Collaborative Innovation Center for Marine Biomass Fibers, Materials and Textiles of Shandong Province, Qingdao University, No. 308 Ningxia Road, Qingdao 266071, China.

^2^Beijing Engineering Research Center for BioNanotechnology & CAS Key Laboratory for Biological Effects of Nanomaterials and Nanosafety, National Center for NanoScience and Technology, 11 BeiYiTiao, ZhongGuanCun, Beijing 100190, China.

*Corresponding author: X.J. (email: [xingyujiang@nanoctr.cn](mailto:xingyujiang@nanoctr.cn)), W. Z. (email: [zhengwf@nanoctr.cn](mailto:zhengwf@nanoctr.cn)), and Y. L. (email: [yunze.long@163.com](mailto:yunze.long@163.com))


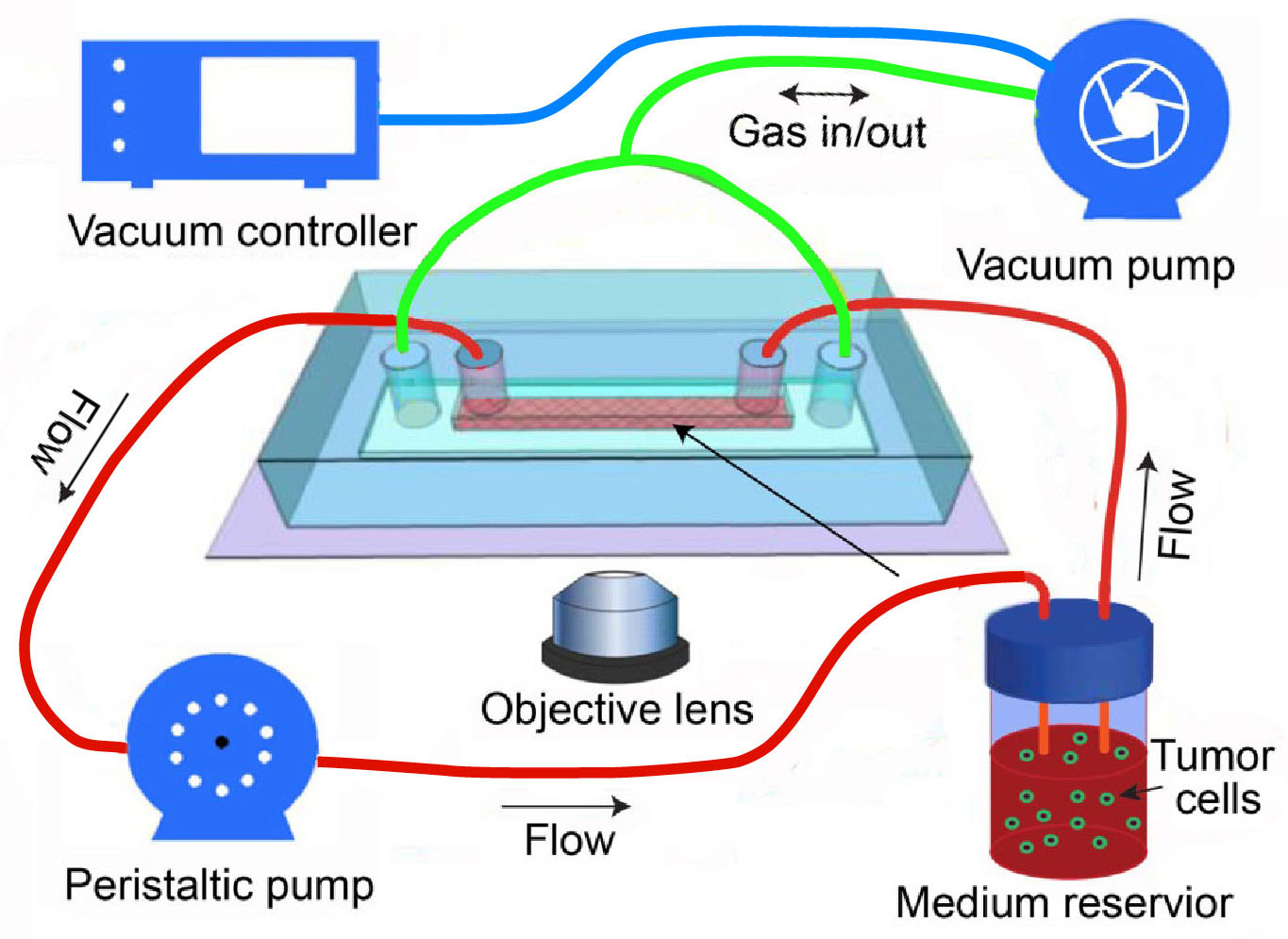


**Figure. S1** A schematic of the components of the whole microfluidic system. The system consists of a microfluidic chip, a flow driving system (peristaltic pump and medium reservoir), and a vacuum generation system (vacuum pump and airflow controller).


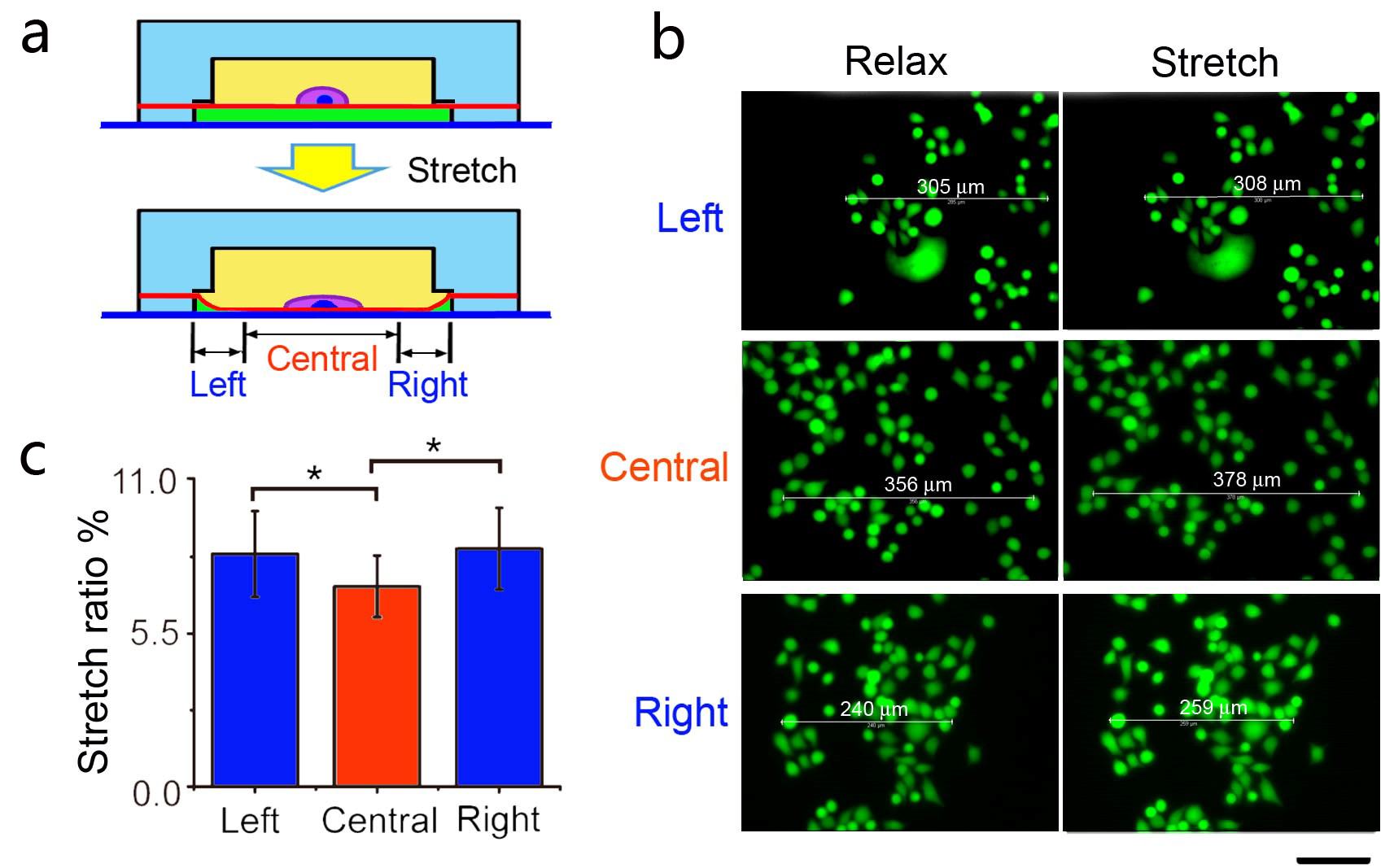


**Figure. S2** The characterization of the stretching property of the elastic membrane. (a) A schematic of the stretching process for the adhered cells. (b) The stretching extents of the membrane at the different areas were quantified by measuring the variation of the distances between the adhered cells. The white lines indicate the distances of the cells. (c) Statistics of the stretching properties of the elastic membrane (*P*< 0.05, Two-Sample *t*-test). Scale bar, 100 μm.


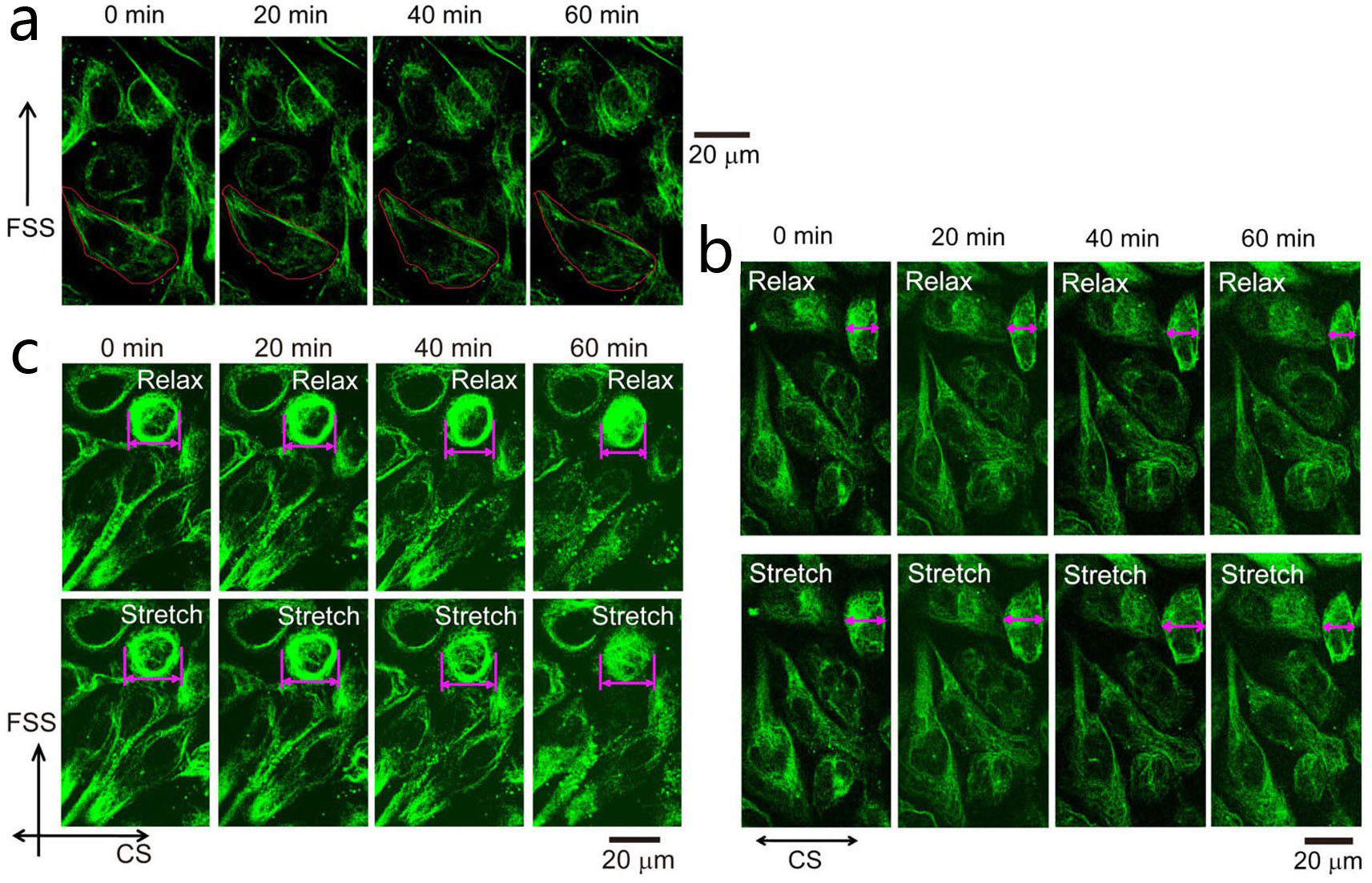


**Figure. S3** Live cell tracking under different mechanical stimulations. (a) Time-lapse images of the dynamics of the cells under FSS stimulation. The single cell outlined by red circle shows dynamic disassembly and reassembly of tubulins. (b) Time-lapse images of the dynamics of the cells under CS stimulation. The pink double arrows indicate the variation of cell size with the deformation of the attached elastic membrane. (c) Time-lapse images shows the effect of combined FSS and CS on the HUVECs. The cells were stained with Tubulin Tracker Green.

**Legends of the movies:**

Movie S1 The real-time imaging shows that the tumor cell floating in the medium moved forward in the direction of the fluid flow, and slowed down its travelling speed and gradually attached onto the substrate.

Movie S2 The real-time imaging shows that some of the tumor cells aggregated and formed cellular clusters which slowed down their speed and attached to the substrate.
